# Microwave Sensors and Their Applications in Permittivity Measurement

**DOI:** 10.3390/s24237696

**Published:** 2024-12-01

**Authors:** Changjun Liu, Chongwei Liao, Yujie Peng, Weixin Zhang, Bo Wu, Peixiang Yang

**Affiliations:** 1 School of Electronics and Information Engineering, Sichuan University, Chengdu 610064, China; 2 Yibin Industrial Technology Research Institute of Sichuan University, Yibin 644000, China; 3 Sichuan Yibin Plastic Packaging Materials Co., Ltd., Yibin 644007, China

**Keywords:** microwave sensor, permittivity measurement, differential signals, metamaterials, microfluidics, cross-technology, algorithms

## Abstract

This paper reviews microwave sensors and their applications in permittivity measurement. The detection, diagnosis, classification, and monitoring without contact and invasion have been the subject of numerous studies based on permittivity characteristics tracking. This review illustrates many new types of research in recent years. Firstly, the application background is briefly introduced, and several main measurement methods are presented. An overview of measurement technology in various applications is compiled and summarized based on numerous typical examples. Exciting applications are compared and presented separately, combining resonator sensors with strong electric fields. Furthermore, differential signals represent trends for future applications with strong environmental immunity, an alternative option to expensive measuring equipment. With the alternation of metamaterials, microfluidics technologies, cross-technology, algorithms, and so on, sensors play an exceptionally prominent role in practical and low-cost applications.

## 1. Introduction

5G, as a key technology field that the country is vigorously developing, has advantages such as high transmission speed, high capacity, and high compatibility. In the field of 5G, microwave devices, as an essential part, are also widely used in other industrial fields [1,2]. In the realization of microwave devices, microwave materials are the foundation and the critical step in development. The change in their electromagnetic properties significantly impacts the performance of microwave devices. Thus, the ability to accurately measure the electromagnetic properties of the materials is of important research significance [3]. 5G technology features low latency, high capacity, faster response times, and the ability to connect multiple devices simultaneously. The monitoring data from these sensors can be uploaded to network terminals in real time. The data can be analyzed and organized to support many innovative technologies, such as remote healthcare and autonomous vehicles. Additionally, with 5G technology, data monitoring from sensors is faster, easier, and more cost-effective [4]. In the field of autonomous vehicles, radar sensors are used to monitor the surrounding environment of the vehicle, which affects its driving behavior. In the study [5], a series-fed slotted coaxial array antenna based on a gap waveguide with multilayer waveguide (MLA) technology was proposed. Its performances with low-cost, high-efficiency characteristics were experimentally verified. These features make the proposed antenna scheme very suitable for application in millimeter-wave automotive radar. In the medical field, wearing micro sensors allows for monitoring patients’ vital indexes, facilitating better remote health monitoring. Ref. [6] proposed a reliable smart earplug sensor based on 5G technology, which was capable of simultaneously monitoring the health of the heart, brain, and lung functions. The recorded data are transmitted via the 5G network, enabling the monitoring of patients’ health conditions.

The measurement of electromagnetic properties of materials mainly includes the measurement of permittivity and magnetic permeability; the industry focuses on the measurement of permittivity. Permittivity is a key parameter used to measure the electromagnetic properties of materials and has important application value in many fields. Among the existing detection methods, microwave detection has gradually become an important means in the field of permittivity measurement because of its advantages, such as high sensitivity, low cost, and simple structure. This paper will review the permittivity microwave sensors and discuss their basic principles, types, applications, and development trends.

## 2. Material Permittivity

The permittivity, as one of the basic parameters reflecting the electromagnetic properties of a material, is a physical quantity describing the ability of a material to store and dissipate energy in an electromagnetic field. The permittivity *ε* comprises the real part *ε*′ and the imaginary part *ε*″, i.e., *ε* = *ε*′ − *j ε*″. The tangent of the loss angle is the ratio between the imaginary and real parts:

(1) $$\tan\delta =\frac{\omega \varepsilon^{\prime \prime }+\sigma }{\omega \varepsilon^{\prime }}$$

where *ε*′ and *ε*″ are the real and imaginary parts of the permittivity, *σ* is the conductivity, ω is the angular frequency of electromagnetic waves, and tan*δ* is the loss angle tangent. For dielectric materials, the *ε*′ is the degree of polarization under the action of an electric field, and the *ε*″ is the electric energy loss in the presence of an electric field. When the value of *ε*′ increases, the ability to store electric energy increases. As a composite indicator, tan*δ* reflects the electric energy loss capability.

Microwaves have different penetration, reflection, and scattering characteristics of the overall materials. Microwave absorption ratio and transmission performance will reflect various materials’ permittivity. For example, microwave sensors may use those characteristics, such as scattering parameters, to obtain material permittivity. Microwave sensors have been successfully applied to various fields.

Therefore, microwave sensors for permittivity measurements are significant in various fields. For example, microwave sensors for permittivity measurements enable non-invasive measurement of blood glucose concentration in the medical field. Masahito Nakamura and others have conducted clinical experiments on non-invasive blood glucose measurement using microwave dielectric spectroscopy and demonstrated the feasibility of microwave sensing for non-invasive glucose measurement [7]. In environmental monitoring, microwave sensors are often used to detect the permittivity of liquids to characterize a specific index. Supa Korn Harnsoongnoen detected the mixed concentration of phosphate and nitrate in liquids, which is important for preventing eutrophication [8]. Maziar Shafiei developed a disposable, low-cost, online microplastic detection device that solves the problem of the traditional method of detecting microplastic particles, which requires a bulky and expensive measuring device [9].

In materials science, especially when developing materials for electronic devices, the electric properties are an important index that cannot be ignored. The material permittivity can reflect the transmission and absorbing performance of microwaves, which are closely related to the material’s mechanical, thermal, and optical properties. Accurate measurement and understanding of permittivity are critical for materials science research and engineering applications. Precise measurement of a material’s permittivity helps drive development in a variety of fields, including electronics, communications, and energy. Therefore, it is crucial to measure a new material’s electric properties accurately. Noor Hasimah Baba scholars proposed a non-contact and non-destructive method to measure the complex permittivity of doped silicon wafers [10], which provides practical assistance in designing high-frequency integrated circuits. It offers effective help for the design of high-frequency integrated circuits. Flexible composite materials (FCMs) are the foundation of wearable devices. The study of the variation of a dielectric constant under different mechanical deformations helps to fully utilize flexibility for circuit design. Ref. [11] proposed a model for predicting the variations of permittivity and anisotropic permittivity under different compressing/stretching ratios and doping concentrations. The reflection and transmission coefficients of building materials, including glass, granite, PVC panels, ceramics, and wood panels, are measured in the indoor wireless communication, which is related to materials’ permittivity [12].

The ability to accurately measure the permittivity is key to the future development of materials, medicine, and other fields. Therefore, it is important to summarize the existing research results. This paper focuses on the classification, applications, and future development trends of microwave sensors for permittivity measurements.

## 3. Permittivity Measurement Sensors

### 3.1. Transmission/Reflection Microwave Sensors

The transmission line method mainly involves placing the Sample Under Test (SUT) into a multi-layer transmission line or directly on the surface of a transmission line. The presence of the samples will change the electromagnetic field distribution around the transmission lines. Thus, variation of the S-parameters of the transmission line will be measured. The S-parameters will inversely infer the permittivity of the SUT [13,14]. 

Due to the transmission characteristics, the permittivity is measured by the transmission line method over a wide band. Thus, transmission/reflection microwave sensors are often used for broadband measurements. Larger measurement errors may be introduced into the measurement due to multipath effects, environmental factors, material placement, and so on.

The low-quality factor is the main problem for the resonators. For the transmission method, reducing the transmission loss will improve the accuracy. Researchers have developed various planar resonators to address this problem. Various methods will be presented in the section on different microwave sensors.

### 3.2. Resonant Cavity Microwave Sensors 

Permittivity is measured by the resonance method through a cubic cavity, a cylindric cavity, or a coaxial resonator. Weak couplings are applied to stimulate electromagnetic waves. Materials are fixed in the resonant cavity. Generally, the electric or magnetic fields inside the cavity are disturbed. Thus, the resonator’s resonant frequency and quality factor will be changed. By solving inverse problems, we obtain the permittivity.

**Figure 1:**
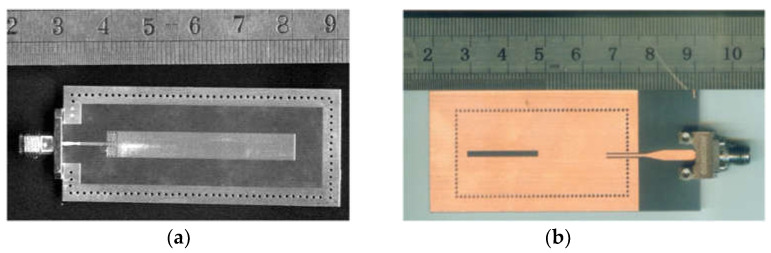
(**a**) Picture of microstrip resonator (from [26]). (**b**) Picture of SIW resonator (from [27]).

Resonant sensors usually have high-quality factors to achieve high measurement accuracy on low-loss materials. Various resonator structures, such as the split ring resonator (SRR) [15,16,17,18], complementary split ring resonator (CSRR) [19,20,21,22], balanced-type circular-disk resonator (BCDR) [23,24], and so on, have been designed for different applications. Three microwave sensors based on the substrate-integrated waveguide (SIW) resonator, which was proposed by Changjun Liu’s team [25,26,27], have combined the sensor with the structure. Later, a microwave sensor based on substrate integrated suspended line (SISL) resonator was designed [28]. In Figure 1a, a λ/2 open-circuit microstrip line excites the resonant electromagnetic field when the array of metal columns prevents energy from escaping. The slot is the test region of liquid samples on the ground. A novel SIW resonator sensor with a high-unloaded quality factor has been designed, as shown in Figure 1b; binary mixtures of ethanol and water are measured rapidly and precisely based on an artificial neural network when the slot is immersed in a liquid sample.

High measurement accuracy, low cost, and small resonator size are the key design indicators for microwave sensors. Compared to other types, a resonator sensor has the advantages of high sensitivity, low cost, and high design flexibility. Thus, microwave resonance-type sensors are the focus of research in permittivity measurement. 

With the development of microwave technology, more and more scholars have made certain breakthroughs in the field of microwave sensors. For example, Yuto Kato et al. found that the resonance frequency shifted by changing the disc size of the BCDR resonator, realizing a stable permittivity measurement of COP samples up to 170 GHz [23]. Hongyi Gan et al. proposed a differential microwave microfluidic sensor based on the microstrip complementary split ring resonator (MCSRR) structure, which eliminated the influence of environmental factors [29]. On the other hand, the resonators usually have specific requirements for the sample shape and size.

### 3.3. Free-Space Microwave Sensors

The free space method uses a pair of antennas for emitting and receiving microwaves and introduces the SUT to interfere with the propagation of electromagnetic waves between the antennas. The SUT’s reflection and transmission affect the propagation of electromagnetic waves. Meanwhile, the phase and amplitude of the electromagnetic wave are used to evaluate the SUT permittivity. The permittivity is inversely deduced by analyzing the S-parameters before and after introducing the SUT. 

The main feature of free-space microwave sensors is contactless detection, making them suitable for the non-destructive evaluation of low-loss dielectric materials. Hassan Shwaykani and others have realized the near-field measurement of relative permittivity using a single golden-tower-shaped horn antenna [30]. However, while most of these methods require the thickness of the SUT to be measured, a new free-space measurement method has been proposed by Scholar Sung Kim et al. to measure the permittivity of low-loss dielectric materials without providing the thickness of the SUT [31]. To ensure that the electromagnetic wave can be maximally incident on the surface of the SUT, the surface of the SUT is often required to be large and flat to ensure that the reflected or transmitted electromagnetic wave is maximally received by the receiving antenna.

## 4. Applications of Microwave Sensors for Permittivity Measurements

### 4.1. Solid Material Application

**Figure 2:**
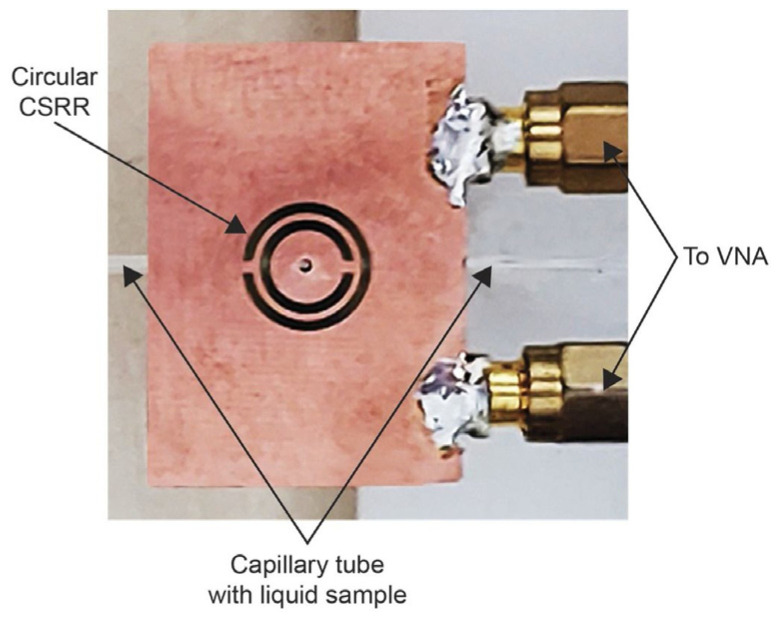
CSRR microwave sensor (from [32]).

Permittivity measurements on various solids and liquids have been well-studied recently. The complementary split-ring resonator (CSRR) structure was applied to the permittivity measurement of liquid materials by etching copper on a dielectric substrate. A planar circular CSRR, as shown in Figure 2, was designed to measure the dielectric properties of liquids. The liquid sample was tested in glass capillary tubes embedded in the substrate perpendicular to the CSRR. The complex permittivity of the samples was estimated when the samples were flowing parallel to the CSRR. Due to the advantages of low cost and integration, the samples of water–ethanol mixtures were measured on an FR4 substrate. By recording the resonant frequency and Q factor, the permittivity of mixtures was reversed at 2.4 GHz [32].

Unlike the CSRR, the SRR and the test area were placed on the same side. Composed of non-identical double-split ring resonators, the microwave sensor was designed to test liquid samples of ethanol, methanol, glucose solution, and deionized (DI) water by being placed in the inner part of the power divider branches. By changing the scale of the two SRRs, the first resonance frequency was from 5.76 to 4.8 GHz, and the second resonance frequency was from 7.85 to 6.35 GHz [33]. Three capacitively loaded periodic slow-wave transmissions were assembled for highly sensitive phase-variation sensors for the permittivity measurements induced by the loading capacitors formed by closely spaced rectangular patches. The material under test (MUT) was measured in the region of the capacitive patches due to modifying the coupling capacitance. According to the phase variation, the phase-variation sensors with high sensitivity and compact size were proposed [34].

For small variations in solute concentration, a planar microstrip-based bridge topology CSRR (B-CSRR) was designed to test several lossy samples incorporated on the top of the sensor region. The topology structure can exhibit a fractional sensitivity of 10.17% and a Q-factor of 170 at 5.18 GHz, which obtains the concentration of solutions by the shift in the resonant frequency related to the permittivity [35]. The dielectric parameter characterization of ethanol liquid samples varied with different ingredient concentrations. Combined with two intercoupled spiral resonators in a band stop filter configuration, the disposable containers were fabricated by 3D printing, which induced the permittivity variations of the Rogers RO4003 substrate at 1.8 GHz [36].

**Figure 3:**
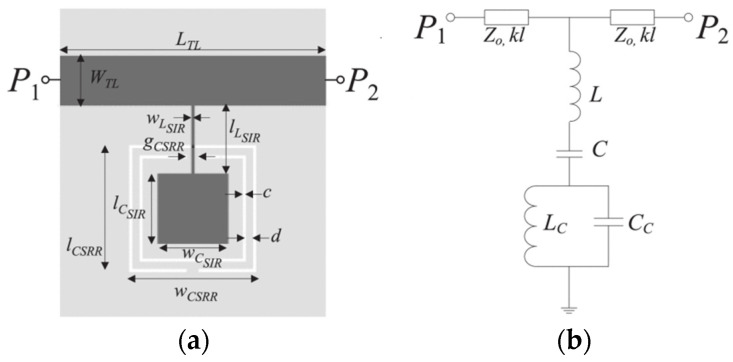
(**a**) Structure with a CSRR-loaded SIR resonator. (**b**) Equivalent circuit model (from [38]).

The CSRR was employed in a novel planar reflective sensor to discriminate different states of vanadium redox solutions at 6 GHz. The dissipated power was offset by an extra loss-compensating negative resistance, which improved the capacity of dielectric characterization when the liquid samples were parallel to the CSRR [37]. A CSRR-loaded step-impedance resonator (SIR) was designed for microfluidic applications and dielectric characterization of solution samples on the underside of the substrate. Figure 3 shows the topology structure of the SIR resonator on the top and a CSRR on the bottom for coupling. According to a controllable pole and two transmission zeros, the volume fractions of 5% isopropanol in DI water can be determined with a maximum measured sensitivity of 4 mV/% [38].

**Figure 4:**
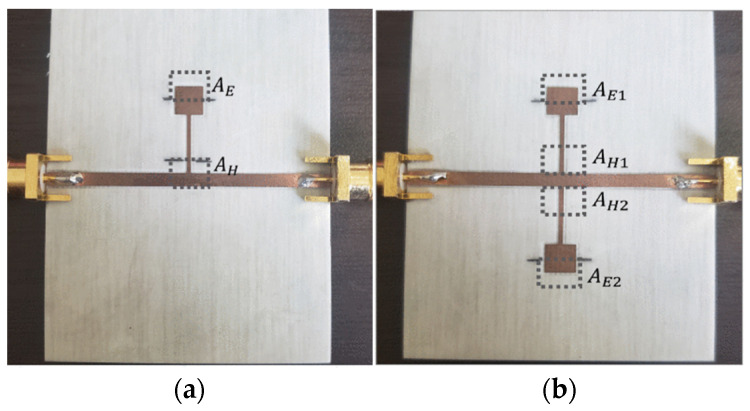
(**a**) The picture of a single SIR (SSIR) sensor and (**b**) The picture of a double SIR (DSIR) sensor (from [41]).

A novel methodology was proposed to design and tune the parameters of the CSRR rapidly to optimize the multi-variable resonators. A super-broad frequency range, from 5 to 20 GHz, is obtained by an analytical correction technique that enables rapid adjustment of geometry parameters. The resonance frequency shift allowed us to determine the permittivity of TLY-5, AD250C, RO4003C, RF-35, and FR4 [39]. After processing by microwave sintering, the lunar regolith can be used for landing pads and building infrastructure. Therefore, the permittivity measurement of lunar simulants, for example, NU-LHT-4M, NUW-LHT-5M, CSM-LHT-1G, and JSC-1A, is performed in the low-temperature range of −185 °C to 250 °C and in the frequency from 50 MHz to 3 GHz, employing the WR340 resonant waveguide cavity to obtain the most energy-efficient processing of regolith [40]. Based on the resonance frequency variation, single SIR (SSIR) and double-step impedance resonance (DSIR) are established to split two and four sensor areas, respectively. Figure 4a shows the areas near the transmission line for the permeability measurements of different substrate materials in the area of *A_H_*. When the permittivity measurement was performed, two regions of rectangular loads in Figure 4b are sensitive to electric field disturbance when the resonance frequency range is 2 GHz to 2.4 GHz [41]. 

The material applications of permittivity measurement sensors are essential in the real-time observation of the dielectric properties of solids and composite fabrications. Thus, recent research mainly focuses on real-time measurement, low cost, and small scale, based on microstrip circuits with the CSRR or SIR methods.

### 4.2. Liquid Material Application

**Figure 5:**
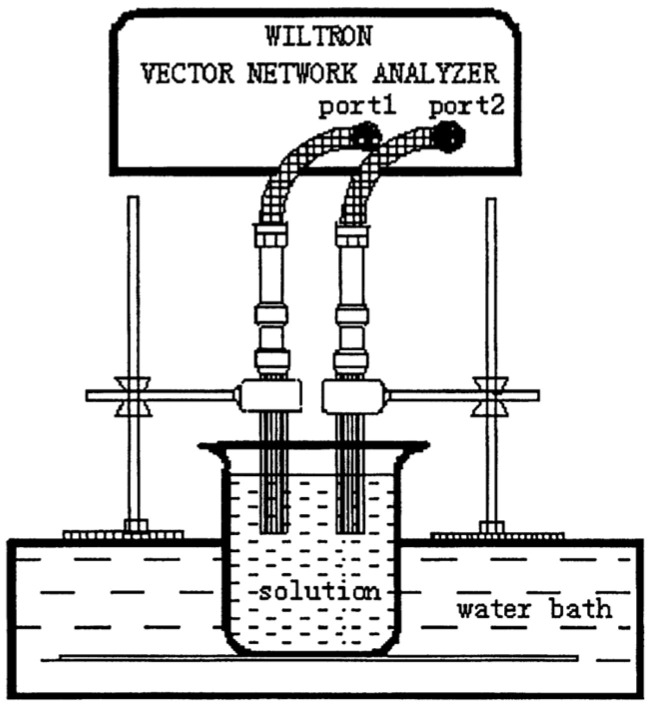
The resonant coaxial sensor system (from [42]).

Various components of a liquid exhibit different dielectric properties. Since complex mixtures composed of saponification reactants change with time, it is necessary to analyze the effective permittivity to achieve the monitoring of the reaction process. Figure 5 shows that a resonant coaxial sensor was designed to attain the reflection coefficient variations in the saponification reaction. Combined with a genetic algorithm-based inverse-calculation technique, the results coincide with Debye’s equation [42].

**Figure 6:**
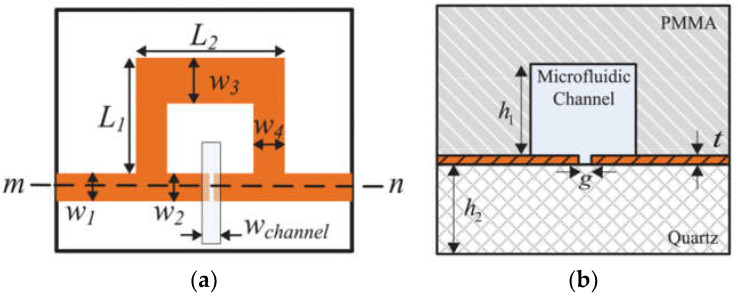
Simulation: (**a**) a top view; (**b**) cross-section view (from [43]).

According to microwave sensors, permittivity measurement allows for environmental monitoring, breaking the limitations of time and space. Figure 6 shows a microwave sensor with the square ring-loaded resonator (SRLR), designed to detect water–caffeine solutions under gradient elution, which is compatible with the HPLC when the microfluidic channel covers the gaps [43]. 

Combined with liquid samples and the CSRR structure, A microwave microfluidic sensor with the particle-ant colony optimization and the wolf pack algorithm allowed for a sensitivity of 0.55% when the microfluidic channel was filled with the mixture of ethanol and water [44]. The variable chemical compositions of liquid samples emerge with different permittivity. A CSRR microwave sensor with capacitive gaps enabled the monitoring of chemical concentrations when the liquid samples were measured in the tube embedded in the CSRR-load groove [45]. Microstrip structures of meander, ladder, and T-structure were applied to measure the ethanal solution, with a sensitivity of 4% and Q-factor of 3500, which was beneficial for monitoring disinfectants [46].

**Figure 7:**
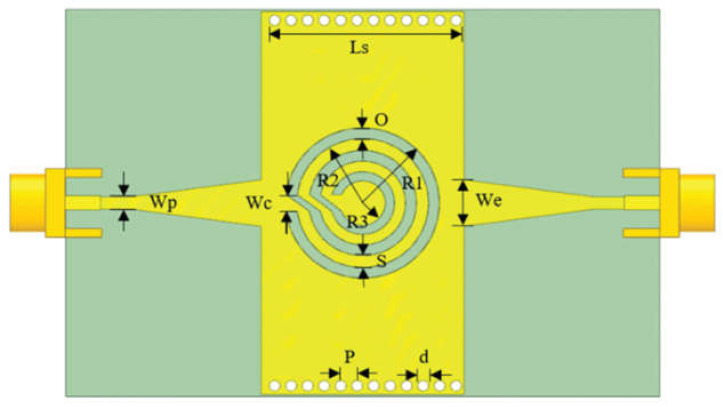
The overall structure of the sensor (from [47]).

As shown in Figure 7, a complementary curved ring resonator (CCRR) is etched based on the substrate-integrated waveguide (SIW) for use in liquid concentration and solution differentiation [47]. *W*_p_ and *W*_e_ are the line width of the microstrip line and the line width of the tapered line, respectively, which influence the impedance match. *R*_1_, *R*_2_, and *R*_3_ denote the radius values of the rings. *O*, *S*, and *W*_c_ are the ring width of the slot, the ring width of the copper ring, and the feeding line width. The values of *P* and *d* affect the transmission loss, which represents the spacing of metal through holes and the diameter of metal through-holes, respectively. Periodically arranged metal through-holes form equivalent electric lengths *L*_s_.

**Figure 8:**
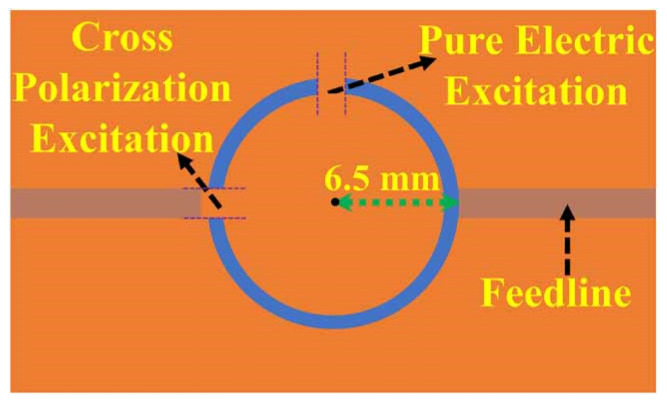
The scheme of split-gap excitations (from [48]).

The microwave sensors are the observation part of the closed-loop control system for liquid applications. The highly integrated and real-time features are significant advantages for continuous industrial production in the industrial production processes. In contrast to the industrial sensors, a microwave sensor with the double split CSRR (DS-CSRR) costs less and possesses dimensions of 20 × 30 mm^2^, possessing the features of low cost and small scale; see Figure 8. The samples, including ethanol, methanol, and chloroform for industrial bulk application, have been tested with less than 3.7% error [48].

**Figure 9:**
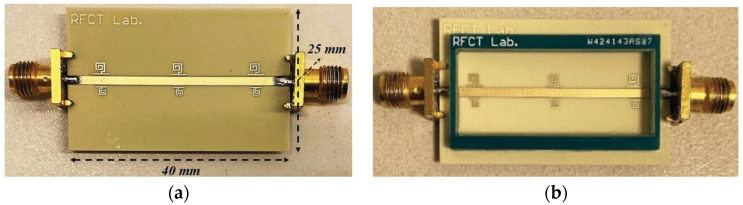
(**a**) Prototype of the sensor. (**b**) The hollow bare FR4 container (from [51]).

The meandered microstrip structure can improve the coupling capacity of electric fields. A microwave meandered sensor tested the industrial liquid samples, including ethanol, methanol, aqueous glucose solution, and deionized water at 6.21 GHz [49]. In the field of producing coal or natural gas, the methanol concentration can be detected by a microwave sensor with an interdigital structure (IDS), chandelier form (CF), meander structure (MS), and T-shaped structure (TSS). The frequency ranges are from 2.14 to 1.46 GHz, with a sensitivity of 2.6% [50]. A spiral resonator excited by an extended horizontal microstrip line (EH-ML) allows for sensitivity improvement when the liquid samples are tested in a specific range of 1.6–2.4 mm. The differential signal ranges from 1.5 to 4.5 GHz to reduce the error to 0.85%, implemented by three modified two-turn rectangular spiral resonators in series connection, as shown in Figure 9 [51]. 

Many studies for liquid applications have applied microstrips to reduce cost and scale, with guaranteed accuracy. Accompanied by the use of microwave sensors, closed-loop control systems will integrate a large number of microwave sensors for liquid real-time monitoring.

Considering liquid properties, the sealed waveguide structure may be the appropriate option for testing. For water quality testing, the open structures of transmission lines can reinforce the electric field for contact measurement. Ref. [52] proposed a novel sealed probe sensor based on a microwave test system for real-time detection and assessment of solid contaminants in natural gas pipelines. Sealed microwave sensors are feasible for detecting small volume fractions of contaminants (less than 0.5%). A new method based on immersion microwave sensors for monitoring trace metals is proposed in [53]. For water affected by mining, a microwave sensor was designed to detect real-time changes in toxic trace metal contamination within the open structures, allowing for direct contact between electromagnetic waves and water samples.

### 4.3. Biomedical Applications

**Figure 10:**
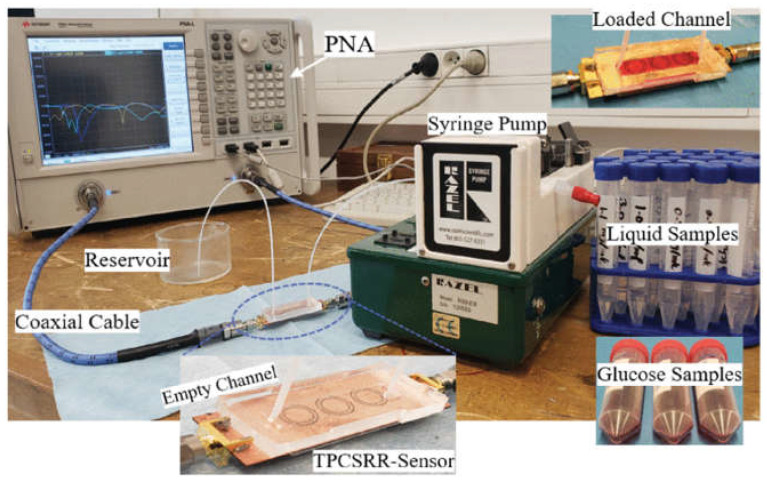
The complete experimental system (from [54]).

Owing to microwave penetration properties, microwave sensors allow for testing the permittivity variations of organisms to detect health and compositional changes. Three CSRRs are cascaded in a microstrip line to reinforce the sensitivity when the microfluidic test region fits snugly with the CSRR. By simulating the blood flowing in the vein, as Figure 10 shows, the sensor capacities for monitoring the varying glucose levels are 33.4 MHz/(mg/mL) and 2.1 dB/(mg/mL) [54]. 

**Figure 11:**
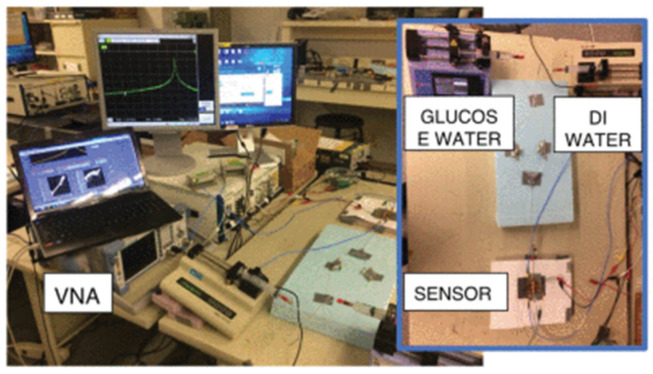
The test system (from [57]).

Considering the application of non-invasive and continuous monitoring, a glucose sensor was composed of a rectangular CSRR for testing and a microwave resonator for the source of the electric field, which enabled a sensitivity 48 times that of the rectangular CSRR [55]. The sensor consists of a microwave ring resonator, which detects the permittivity variations with Escherichia coli (E. coli) growth. The amplitude variation curves are 0.08 and 0.13 dB/h for 3 µL and 9 µL, respectively, similar to the Gompertz growth model. To balance between small scale and maintaining the effective low-loss characteristics of the sample encapsulated in a Petri dish, the resonant frequency was chosen to be 1.76 GHz [56]. A high Q-factor microwave sensor was designed to test the glucose concentration based on the coupling effect. The system in Figure 11 for active ingredient detection, composed of a split ring resonator (SSR) for loss compensation, allows for the lowest glucose level of 1 mMol [57]. 

**Figure 12:**
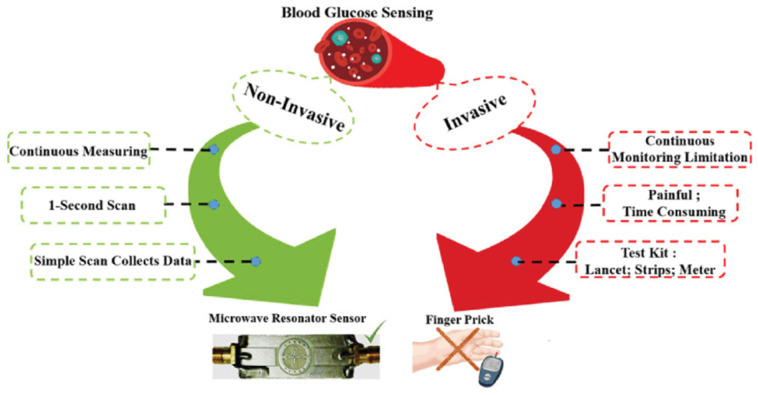
Comparison between invasive and noninvasive measurements (from [58]).

**Figure 13:**
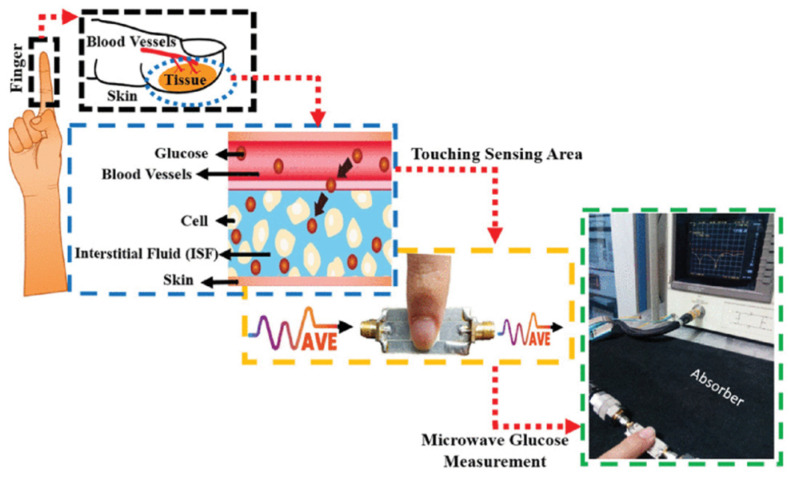
Finger tissue model and test system (from [58]).

As Figure 12 shows, traditional invasive methods for blood glucose sensing will cause discomfort to the examined person, trading off noninvasive measures for convenience. Based on the SIW and coplanar waveguide (CPW) structure, a noninvasive glucose resonance sensor was proposed, as shown in Figure 13. When the finger was placed in the sensing area, the glucose concentration in the blood vessels was monitored via the test system. The black box and the blue box are biological tissue pictures. The yellow and the green box are the sensor system. The circuit consists of the SIW cavity, CPW feed, and sensing area, detecting the frequency variations of 106.11 and 89.262 MHz/(mg/dL), respectively, at 5.5 GHz and 8.5 GHz [58]. 

**Figure 14:**
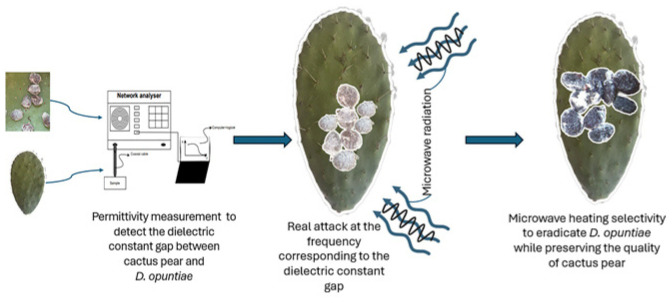
Industrial application in Morocco and coaxial probe for permittivity measurements (from [60]).

Combined with the SIW and the coupling between two resonators, two non-contact test methods with horizontal placement and vertical placement allowed the pharmaceutical industry to reduce errors to less than 3% [59]. Figure 14 shows that *Dactylopius opuntiae* (*D. opuntiae*) creates a considerable threat to cactus plantations globally. According to the permittivity of *D. opuntiae*, the microwave detective method enables it to be chemical-free, with no environmental impact by controlling the power and time, which shows the potential of microwave pest prevention without side effects [60]. 

Surface plasma polarization (SPP) excitations were applied to differential dielectric sensors with the CPW. The differential splitter facilitates observing the relative phase difference in the THz frequency region [61]. The biomedical applications of microwave sensors present non-invasive and real-time characteristics. Using the CSRR, CPW, SSR, and SPP, changes in the composition of human blood can be detected by the coupling of the skin. In the future, wearables may be an alternative to medical devices through microwave sensors.

## 5. Developments and Challenges

### 5.1. Technology Development Trends

**Figure 15:**
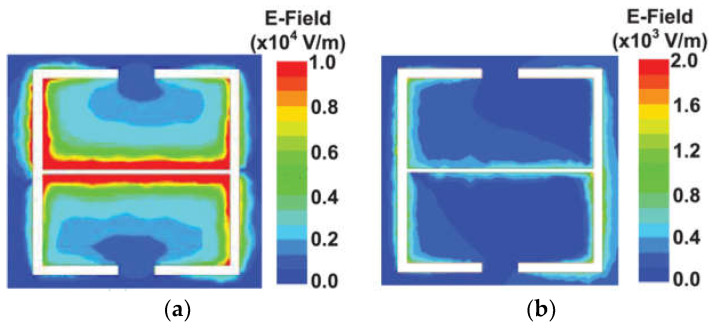
Distributions of (**a**) odd-mode electric fields and (**b**) even-mode electric fields (from [62]).

The differential method, via a comparison between the reference and test frequencies, can suppress the effects of environmental factors. The distributions of different electric fields are shown in Figure 15. Leveraging the magnetic-LC (MLC), a splitter/combiner was developed by separately etching two MLC resonators asymmetrically, which was calculated and measured to make the sensitivities of the odd and even mode reach 2.21% and 1.13% [62]. When the electric field is in the odd mode, the maximum upper limit of the E-field is 10 × 10^3^ V/m. For the even mode, the maximum upper limit of the E-field is 2 × 10^3^ V/m, which is five times smaller than the odd mode.

**Figure 16:**
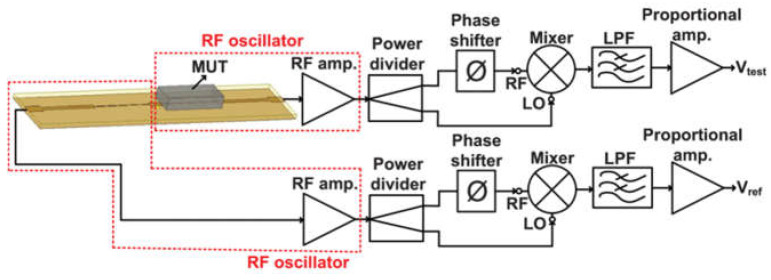
Diagram schematic of the differential sensing system (from [65]).

Unlike the upper and lower split structure, two circular spiral resonators (CSRs) were equally etched on both sides of the microstrip line. The sensitivity was calculated to be about 0.28% [63]. The combinations of the SIW and the CSRR can improve the coupling effect of the electric field. Based on the differential method, two capillary glass tubes, as sample containers, were applied vertically through the dielectric substrate, for a measurement sensitivity of 8.2 MHz/(Δε_r_·μL) [64]. The binary relationship between the actual permittivity and the oscillation frequency is converted to a relationship between the actual permittivity and the output DC voltage by combining a symmetric passive stepped impedance and two oscillators. A differential active microwave sensor was developed based on the step-impedance transmission lines, a low-cost alternative to the VNA, with a maximum error of 1.745%, as shown in Figure 16 [65].

Metamaterials are heavily investigated as a means of sensor sensitivity enhancement. Due to superior dielectric properties, three double-slit complementary split rectangular resonators were employed for the metamaterial sensor, which has a lower error of 0.77% [66]. As the anisotropic material, the permittivity of liquid crystals (LCs) varied with the frequency variations. A waveguide was designed using the epsilon-near-zero (ENZ) metamaterials for accurate measurement. The enhanced electric field surrounded the test region under the action of metamaterials [67].

**Figure 17:**
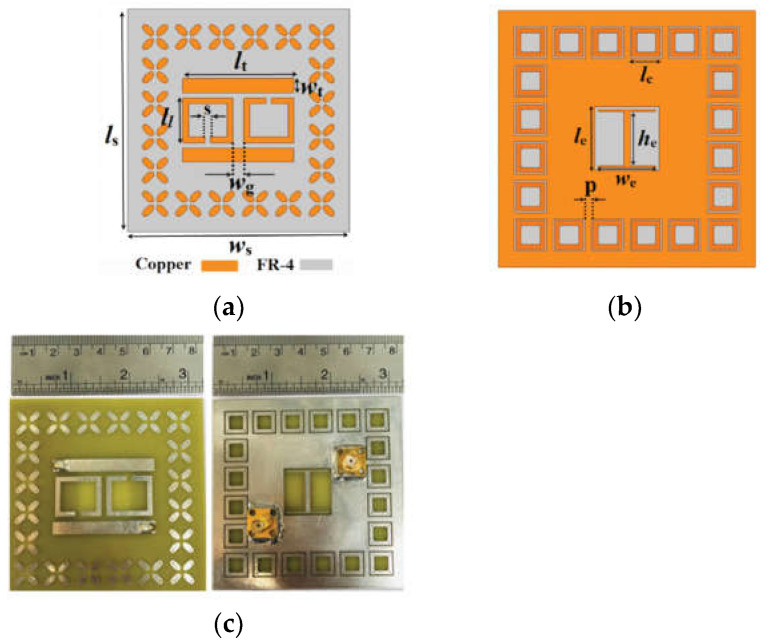
Schematics of the sensor: (**a**) top view, (**b**) ground layer, (**c**) practical fabrication (from [69]).

Microfluidics technology enables it to be applied in the field of liquid measurement, with precise control of the volume. Using a sensing oscillator with a modified coplanar strip resonator (MCSR), samples filled with the microfluidic tube and the coupling region between the strip line and the coplanar ground were tested. The maximum errors were 9.45% and 8.84% for the real and loss tangent parts, respectively [68]. As shown in Figure 17, two coupled split-ring resonators (SRRs), including the sensing region, are surrounded by a cycle of electromagnetic band gap (EBG) unit cells used as metasurfaces to enhance the electric field. The sensitivity of FR-4 substrates with photonic band gap (PBG) properties is around 4.68% [69]. 

**Figure 18:**
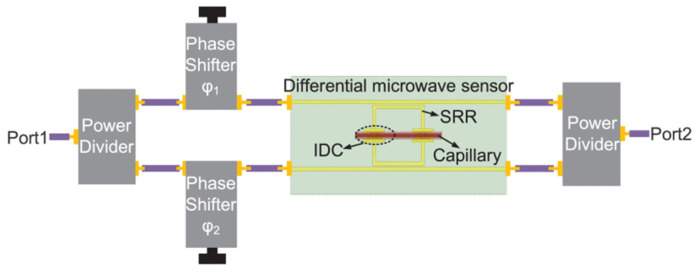
Schematic of the measurement system (from [71]).

A novel CSRR sensor combined with metamaterial with added extension and curved notch was used for the electric field confinement. The sensor exhibited sensitivities of 4.12% and 0.78% for solid and liquid samples, respectively, which are smaller than in works [68,69]. The results show that the sensor is characterized by high sensitivity, high measurement accuracy, low cost, and easy fabrication [70]. The differential passive sensor composed of two split-ring resonators (SRRs), interdigital capacitances (IDCs), and four 3D metallic walls is shown in Figure 18. When two common-mode (CM) suppression-based signals are inputted into the sensor with a phase difference of 180°, the information on the permittivity variations is extracted from the variations of relative frequency shift and normalized Q. As established by measurement, the sensitivity is about 0.05% [71]. 

**Figure 19:**
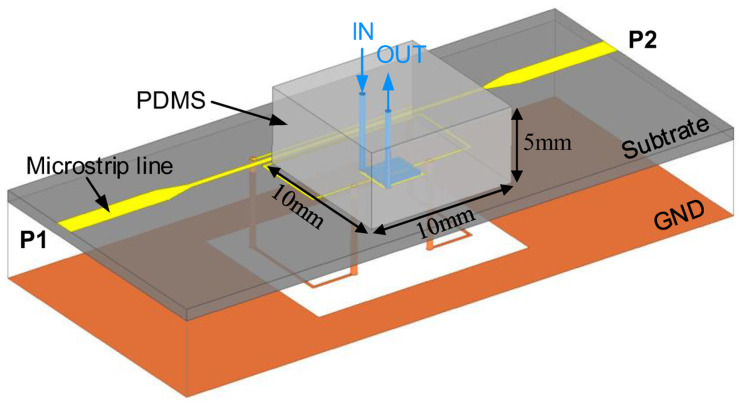
Schematic of the DGS-IDC-DSRR-based microfluidic sensor (from [72]).

The microfluidic method reduces the error caused by the gap between the sensor and the samples. Based on the DGS, IDC, and double SRR technologies, a microfluidic microwave sensor with ultrahigh sensitivity, as shown in Figure 19, was designed and tested for when the channel route coincides with the interdigital gap [72]. 

**Figure 20:**
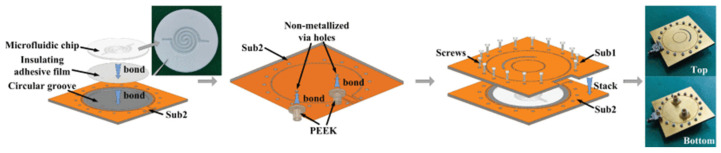
Structure of the microfluidic antenna sensor (from [75]).

**Figure 21:**
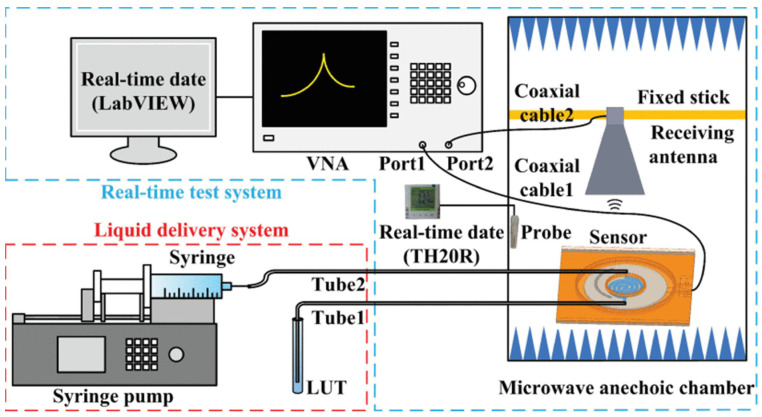
The test system schematics (from [75]).

**Figure 22:**
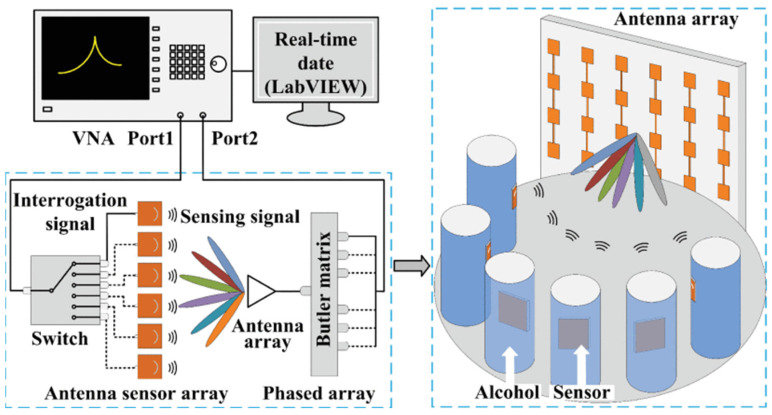
Sketch of the wireless sensing network (from [75]).

The errors triggered by the contactless measurement were solved through a microfluidic differential sensor that consisted of an interdigitated-electrode (IDE) structure and a gain/phase detector (GPD). The maximum errors of the real and imaginary parts are 8.28% and 14%, respectively, calculated by the corresponding magnitude voltage and phase voltage without a VNA [73]. The embodied container was applied in the sensor with two symmetrically positioned interconnected SRRs, which can be regarded as the perturbation of the equivalent permittivity of the substrate [74]. Due to the spatial attenuation, the antenna for the contactless measurement has an inevitable system error. Leveraging a cavity-backed slot antenna (CBSA) and a substrate-integrated waveguide re-entrant cavity resonator (SIW RECR), Figure 20 shows that the slot antenna radiated the corresponding signal affected by the microfluidics embedded in the SIW. The maximum error of the real was 3.5%, with 5.63% for the imaginary one, which was inversed by the resonant frequency shift and transmission amplitude offset. The interesting system for single unit testing and monitoring multiple target objects is shown in Figure 21 and Figure 22, respectively [75].

The combination of metamaterials and microfluidics improves integration and precision. The high electric field coupled by a modified square split-ring resonator (SRR) and a microstrip transmission line (MTL) was applied to detect a change of 1 mg/dL glucose solution in the microfluidic tube with a maximum error of 0.7%, which was obtained by comparing a second-order polynomial [76]. Compared with the previous works, the microfluidic tube integrated the test region into the substrate, which reduced the measurement errors due to poor contact. In reference [76], the maximum error of the microfluidic sensor is 0.7%, which is less than the maximum error of 3.5 and 5.63% in [75].

### 5.2. Future Challenges 

**Figure 23:**
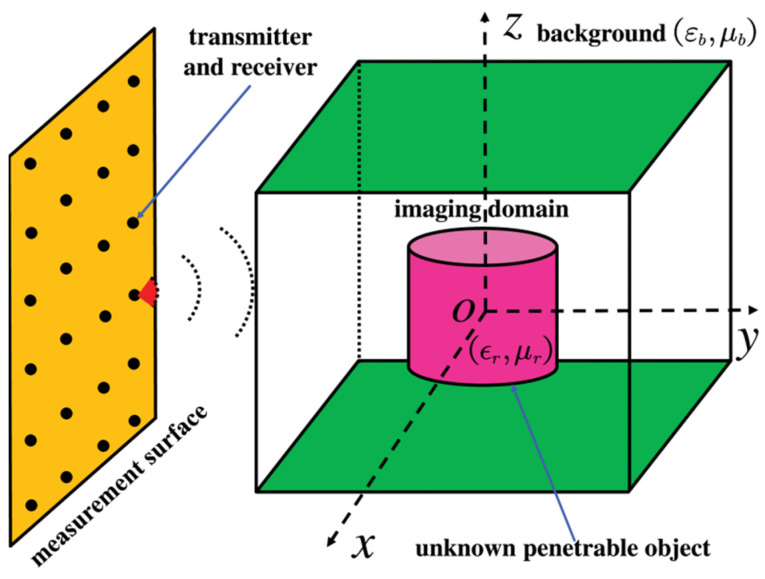
The reconstruction measurement setup (from [78]).

The materials’ permittivity is related to the temperature and the operating frequency. The thermal runaway may emerge in microwave drying and heating with the temperature increase. Therefore, the real-time monitoring system and the calibration algorithm are critical for biomedical, chemical, and industrial applications. Based on the oblique aperture ridge waveguide and the neural network, a system with a rapid response allows for the permittivity measurement of methanol and ethanol during heating, which is rewarding for the applications of microwave energy in the chemical industry [77]. The penetrable characteristic is a unique advantage of microwave illumination. In Figure 23, a reconstruction method is proposed for the permittivity and permeability distribution of mineral substances, applying the Nyström method combined with the multilevel fast multipole algorithm (MLFMA) as well as a Gauss–Newton minimization method (GNMM) combined with a multiplicative regularization scheme (MRS) to solve the inverse problem [78]. 

**Figure 24:**
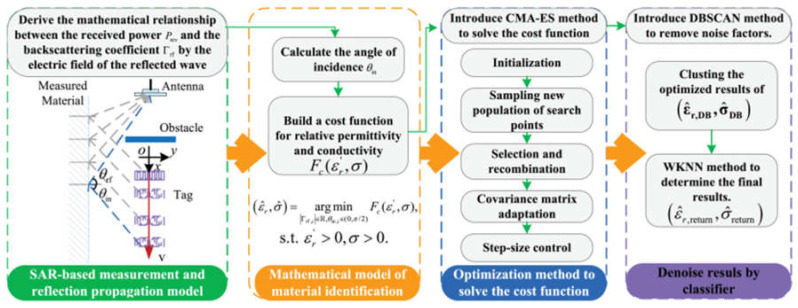
The DIMAR algorithm (from [81]).

Permittivity is an essential design parameter for reducing costs. In the V band, the reconstruction method induced a time-gating procedure to shift the ringing to frequencies based on the Baker-Jarvis algorithm, which can analyze a broader range of initial start optimizations with unknown sample thickness [79]. Tiny defects in composites detected by microwave non-destructive testing (NDT) were implemented based on the distorted born iterative method (DBIM). With the electric field variations, a multi-parameter inversion method based on the dyadic green function of layered media can reconstruct the distribution [80]. Contactless sensing can break through the space barrier to identify the permittivity of different materials on the Internet of Things (IoT). Using radio frequency identification (RFID), the DIMAR algorithm, as shown in Figure 24, leverages blocking the line-of-sight (LOS) between the reader and tag and from the perspective of electric fields, which also combines with the covariance matrix adaptation evolution strategy (CMA-ES) and density-based spatial clustering of applications with noise (DBSCAN) algorithm to provide a solution, even with environmental distractions [81].

**Figure 25:**
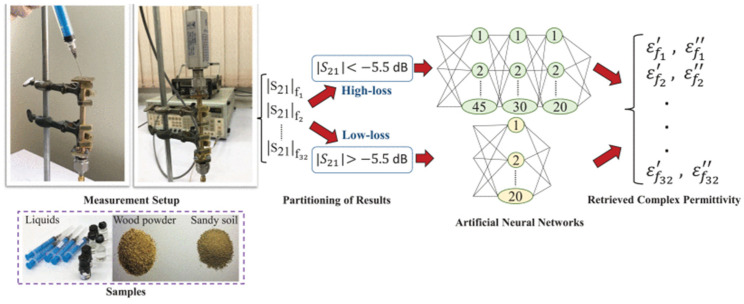
The MLA system and artificial neural networks (from [82]).

Traditional methods relied on the S-parameters extracted by the network analyzer to observe dispersion and amplitude information. A machine learning-aided (MLA) method was proposed to inverse the reflection power, which only depends on simple coaxial and power sensors. The permittivity characterization procedure is shown in Figure 25 [82].

**Figure 26:**
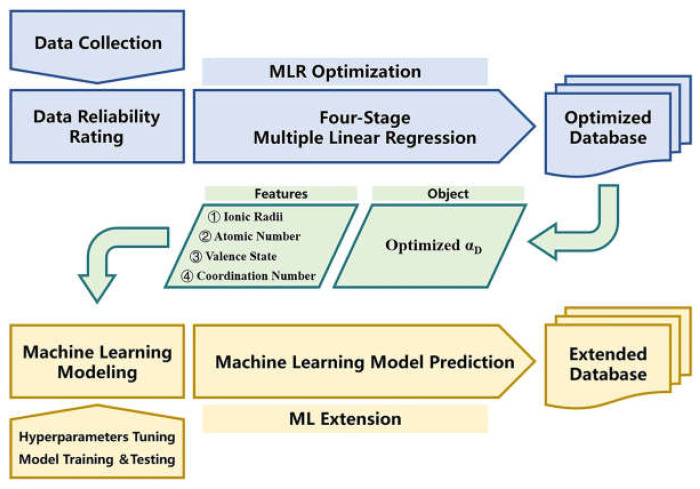
The procedures of the MLR optimization and ML extension of the ion dielectric polarizability database (from [83]).

Due to the permittivity variations of ceramics correlated to frequency, the most influential α_D_ database put forward by Shannon makes it challenging to meet high-frequency design requirements. An interesting perspective for permittivity inversion on the database is shown in Figure 26. Leveraging the four-stage multiple linear regression and support vector machine model, the optimized and extended databases can be applied to passive device selection, including antennas, filters, resonators, capacitors, etc. [83]. 

**Figure 27:**
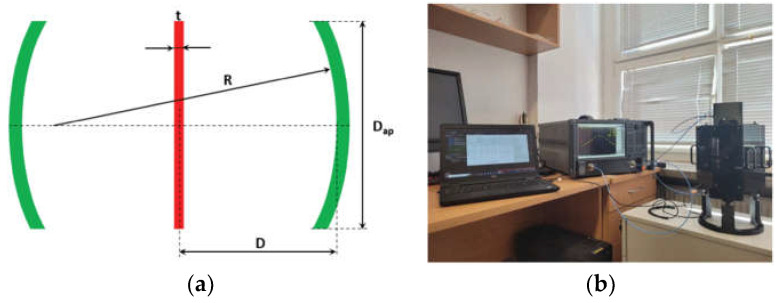
(**a**) Schematic of a DC FPOR and (**b**) measurement setup (from [85]).

A genetic algorithm (GA) was employed to retrieve the buried object through ultra-wideband (UWB) radar technology to acquire the online geological conditions of a tunnel face. After validation, the results show a maximum error of 14% and a 2.5 mm absolute error [84]. A Fabry–Perot open resonator (FPOR) was used to extract the S-parameters multiple times in the microwave and millimeter-wave range. The schematic and system are shown in Figure 27. According to the aid of curve-fitting algorithms and the uncertainty bars of thickness variations, systematic and random errors mainly caused by the electric energy filling factor error and Q-factor variation were evaluated and suppressed [85]. 

A large amount of power may weaken reflective features measured in reflection coefficient values using an open-ended coaxial probe (OECP) for the thin sample measurement. The genetic algorithm (GA) and full-wave numerical simulations show the potential of ultrathin measurement [86]. The reconstruction accuracy for complex permittivity profiles is the conversion of dielectric measurements from point to surface. The multilayer perceptron (MLP) neural network (NN) implemented a reconstruction of the complex permittivity three-dimensional profile, which allows for the reduction of the skin surface rejection [87]. Considering the design and development of microwave dielectric ceramics (MWDC), the spinel permittivity database was constructed for the prediction. Compared to several algorithms, the extreme gradient boosting (XGBoost) model achieved an R-squared (R2) of 0.9095, a mean absolute error of 1.02, and a root mean square error of 1.96, which is the highest degree of fit [88]. The deployment of IoT-5G infrastructures or next-generation communications systems depends on the permittivity of coals. The microstrip-type circuit was designed on the substrate of coals, which was applied with inversion using the Nicolson–Ross–Weir algorithm [89].

**Figure 28:**
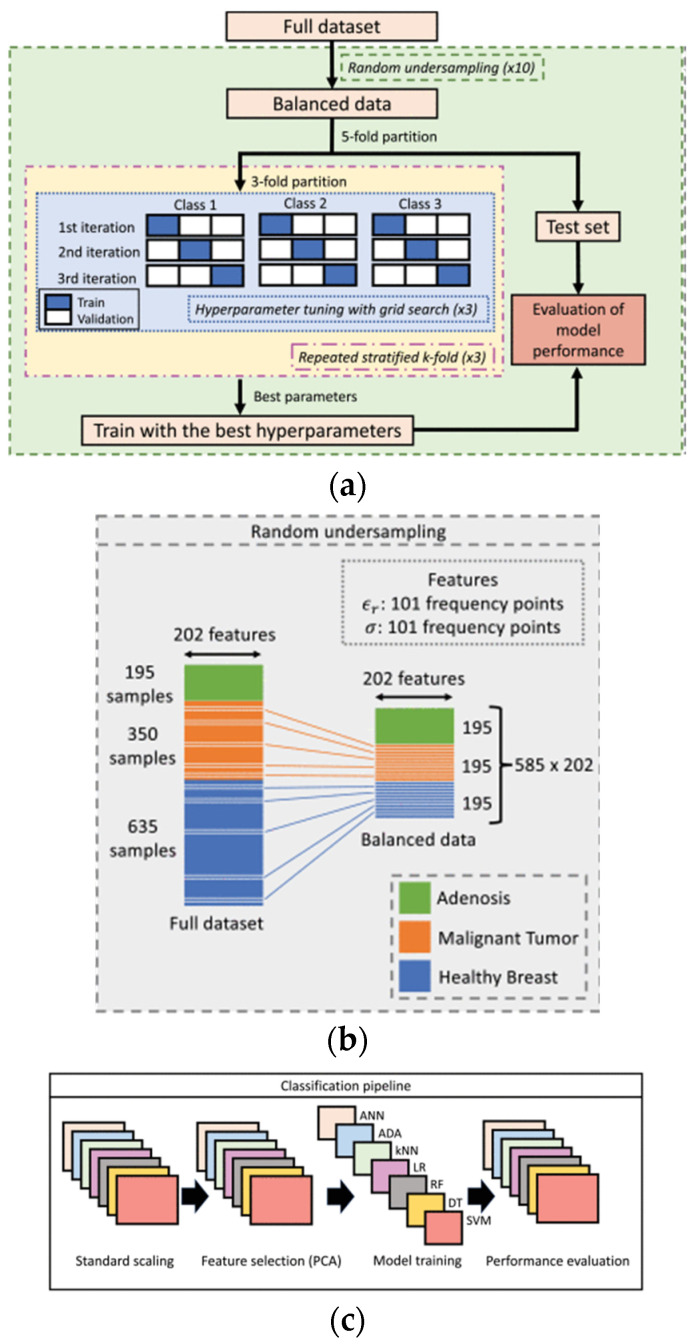
(**a**) The dataset. (**b**) Undersampled data. (**c**) Each ML model (from [90]).

The muscle analyzer system (MAS) used a band-stop filter with several resonators. Figure 28 illustrates the data preparation and ML application processes. Except for the ineffective muscle inversion, the three-stage algorithm was superior in skin and fat predictions [81]. Microwave diagnostics relied on measuring tissue dielectric properties (DPs). For biopsy procedures, Cole-Cole parameters extracted from the open-ended coaxial probe (OECP) technique were classified by the support vector machine (SVM) algorithm [90]. 

A low-cost sensor with an artificial neural network (ANN) was developed, which consists of a modified microstrip line with an interdigital structure (IDS) and a passive part as the alternative to the expensive VNA. The network between complex permittivity and DC voltages was established and resulted in average sensitivities of about 2.478 mV/ε_r_’ and 1.418 mV/ε_r_ for channel-I and channel-Q [91]. Hence, the algorithm for inversion, calibration, and database creation is a primary methodology for enhancing sensitivities instead of optimizing sensor structures. With the convergence of electromagnetic coupling effects, the errors of the non-invasive contactless application can be offset by collecting more parameters.

## 6. Conclusions

Microwave sensors for permittivity measurements are promising for various applications in industrial fields. This paper introduced in-depth reviews of state-of-the-art microwave sensors for measurements and monitoring, highlighting relevant methods and advantages across various applications. With continuous technological advancement and innovation, microwave sensors will achieve higher measurement accuracy and sensitivity, providing a higher integration and lower cost in materials science, environmental monitoring, and biomedicine. Meanwhile, rapid responses depend on a stronger electric field intensity through metamaterials or resonators and more efficient algorithms through differential signals or microfluidics to promote the continuous development of microwave sensor technology. By using the microfluidic method, the error data can be reduced to a range from 3 % to 15%. For the differential method, the error data can be constrained to 3% or less. Further, combined with the metamaterial method, the error data can be further diminished to within 1%. Assuming that multi-component dielectric constant image reconstruction is performed, algorithms and databases are essential steps to differentiate between different dielectric responses.

The following are some of the most promising permittivity measurement methods for future application: (1) reducing systematic errors in the liquid to be measured by microfluidics., which may be applied in biomedical or medical fields to measure/monitor health indexes; (2) offering resonator-based metamaterial for enhancing the electric field strength, which will enhance the sensitivity of microwave sensors and measure permittivity more accurately; (3) based on various resonators, leveraging the differential signals to weaken the environmental impact, which is crucial for practical applications to avoid environmental noise and disturbance; (4) combined with deep learning, the database or the fitting network is established for accurately solving the inverse problem to obtain permittivity. With the fast development of AI and deep learning, obtaining permittivity from measured data of microwave sensors will be more convenient and accurate.

Microwave sensors for permittivity measurements will be applied in various fields to sense the variation in material characteristics. They may be found in more industrial applications and will play an important role in intelligent sensing.

## Data Availability

The original contributions presented in the study are included in the article; further inquiries can be directed to the corresponding author.
